# Factor XI antagonists: the discovery of the philosopher's stone?

**DOI:** 10.1093/ckj/sfae106

**Published:** 2024-04-22

**Authors:** An S De Vriese, Nóra Ledó

**Affiliations:** Division of Nephrology and Infectious Diseases, AZ Sint-Jan Brugge, Brugge, Belgium; Department of Internal Medicine, Ghent University, Ghent, Belgium; Department of Internal Medicine and Oncology, Semmelweis University, Budapest, Hungary

**Figure 1:**
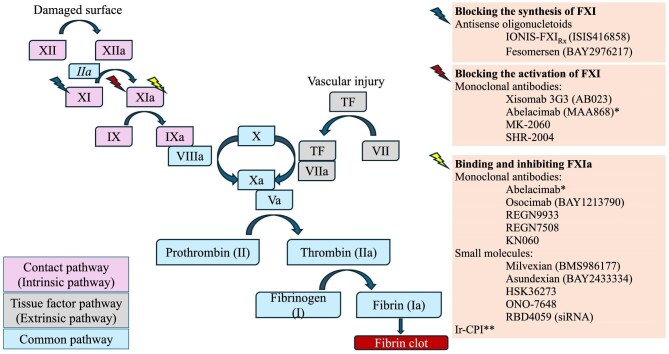
FXI is part of the intrinsic/contact pathway of coagulation and is activated by FXIIa. Its activation is enhanced by thrombin (FIIa), as part of a feedback loop. There are three main types of FXI inhibitors: antisense oligonucleotides that bind to FXI mRNA and catalyse its degradation, thus reducing the synthesis of FXI in the liver; monoclonal antibodies that bind to FXI and block its activation by FXIIa; and monoclonal antibodies or small molecules that bind and inhibit FXIa. *Abelacimab inhibits both FXI and FXIa. **Ir-CPI (*Ixodes ricinus* contact phase inhibitor) inhibits both FXIa and FXIIa.

The widely held belief that thrombosis and bleeding are the opposite sides of the same coin has been challenged by research on factor XI (FXI) inhibition. The role of FXI within the coagulation pathway is primarily supportive: it is responsible for thrombus growth and stabilization by amplifying thrombin formation and enhancing clot resistance to fibrinolysis, but it is not essential for clot formation in response to tissue injury (Fig. 1). Inhibition of FXI may thus prevent pathological thrombus propagation without interfering with normal haemostasis. Several oral and parenteral FXI/XIa inhibitors have completed phase 2 studies in different settings and fulfilled the promise of low bleeding complications (Table 1). Phase 3 trials in cancer-associated thrombosis (NCT05171049, NCT05171075) and stroke (NCT0586070) are ongoing. One phase 3 trial in atrial fibrillation (AF) was interrupted prematurely for inferior efficacy versus apixaban [1].

**Table 1:** Phase 2 trials of FXI inhibitors.

Drug study	Population (*N*)	Compared agents	Safety	Efficacy	Results
**Orthopaedic surgery**					
Abelacimab (EudraCT 2019-003756-37)	Unilateral total knee arthroplasty (412)	Abelacimab 30 mg, 75 mg, 150 mg Enoxaparin	No significant difference in major or clinically relevant nonmajor bleeding	30 mg abelacimab was non-inferior to enoxaparin; 75 mg and 150 mg abelacimab was superior to enoxaparin	doi: 10.1056/NEJMoa2105872
IONIS-FXI_Rx_ (NCT01713361)	Unilateral total knee arthroplasty (315)	IONIS-FXI_Rx_ 200 mg, 300 mg Enoxaparin	No significant difference in major or clinically relevant non-major bleeding	No significant difference in venous thromboembolism	doi: 10.1056/NEJMoa1405760
Milvexian (NCT03891524)	Unilateral total knee arthroplasty (1242)	Milvexian 25 mg, 50 mg, 100 mg, 200 mg Enoxaparin	No significant difference in major or clinically relevant non-major bleeding	No significant difference in venous thromboembolism	doi: 10.1056/NEJMoa2113194
Osocimab (NCT03276143)	Unilateral total knee arthroplasty (813)	Osocimab Enoxaparin Apixaban	No difference in serious adverse events	Postoperative osocimab 0.6 mg/kg, 1.2 mg/kg and 1.8 mg/kg was non-inferior and preoperative 1.8 mg/kg was superior to enoxaparin for venous thromboembolism	doi: 10.1001/jama.2019.20687
KN060 (NCT06180889)	Unilateral total knee arthroplasty (240)	KN060 Enoxaparin			Ongoing
REGN9933 (NCT05618808)	>50 years, unilateral total knee arthroplasty (373)	REGN9933 Enoxaparin Apixaban			Ongoing
**Atrial fibrillation, stroke, myocardial infarction**					
Abelacimab (NCT0421380)7	Atrial fibrillation (28)	Abelacimab 120 mg, 180 mg Placebo	No significant difference in clinically relevant bleeding		doi: 10.1111/jth.15577
Asundexian (NCT04218266)	Atrial fibrillation (755)	Asundexian Apixaban Placebo	Lower rates of bleeding with asundexian compared to apixaban		doi: 10.1016/S0140-6736(22)00456-1
Asundexian (NCT04304534)	Acute myocardial infarction (1601)	Asundexian Placebo	No significant difference in major or clinically relevant nonmajor bleeding	Dose-dependent inhibition of FXIa activity	doi: 10.1161/CIRCULATIONAHA.122.061612
Asundexian (NCT04304508)	Ischaemic stroke (1808)	Asundexian Placebo	No significant difference in major or clinically relevant nonmajor bleeding	No reduction of the composite of covert brain infarction or ischaemic stroke	doi: 10.1016/S0140-6736(22)01588-4
Milvexian (NCT03766581)	>40 years, acute ischaemic stroke or transient ischaemic attack (2366)	Milvexian 25 mg, 50 mg, 100 mg, 200 mg twice a day Placebo Clopidogrel Aspirin	Increase in major bleeding events at doses of 50 mg twice daily and higher	No reduction of symptomatic ischaemic stroke and incident covert brain infarction, numerically fewer symptomatic ischaemic strokes than placebo	doi: 10.1016/S1474-4422(23)00403-9
Abelacimab (NCT04755283)	>55 years, atrial fibrillation (1200)	Abelacimab Rivaroxaban			Ongoing
Ir-CPI (NCT05970224)	Spontaneous intracerebral haemorrhage (32)	Proof-of-concept study			Ongoing
**Haemodialysis**					
Fesomersen (NCT04534114)	Haemodialysis (307)	Fesomersen 40 mg, 80 mg, 120 mg Placebo	No significant difference in major or clinically relevant non-major bleeding	Dose-dependent reduction of FXI levels associated with reductions in haemodialysis circuit clotting and AV access thrombosis	doi: 10.1016/j.kint.2024.02.024
IONIS-FXI_Rx_ (NCT03358030)	Haemodialysis (213)	IONIS-FXI_Rx_ 200 mg, 250 mg, 300 mg Placebo	No difference in major or clinically relevant non-major bleeding		clinicaltrials.gov
IONIS-FXI_Rx_ (NCT02553889)	Haemodialysis (49)	IONIS-FXI_Rx_ 200 mg, 300 mg Placebo	No significant difference in major or clinically relevant non-major bleeding		doi: 10.1016/j.ekir.2021.11.011
Xisomab 3G3 (NCT03612856)	Haemodialysis (24)	Xisomab 3G3 Placebo	No significant difference in major or clinically relevant non-major bleeding	Reduced intradialyzer clotting during heparin-free haemodialysis	doi: 10.1182/blood.2021011725
Osocimab (NCT04523220)	Haemodialysis (704)	Osocimab Placebo	No significant difference in major or clinically relevant non-major bleeding	Lower risk of clotting of the dialysis circuit	doi: 10.1038/s41591-023-02794-7
MK-2060 (NCT05027074)	Haemodialysis with arteriovenous graft (489)	MK-2060 Placebo			Ongoing
**Various**					
REGN9933 and REGN7508 (NCT06299111)	Peripherally inserted central catheter (195)	REGN9933 REGN7508 Placebo			Ongoing

A drug that could effectively reduce the rate of thrombotic events without simultaneously increasing the risk of bleeding would be particularly useful in patients with end-stage kidney disease (ESKD), a population characterized by a high burden of cardiovascular disease as well as a severely elevated bleeding risk. The CONVERT trial is a multinational phase 2b randomized, double-blind, placebo-controlled trial examining the inhibitory FXIa antibody osocimab in 704 patients with kidney failure undergoing haemodialysis (HD) [2]. While patients with a pre-existing high bleeding risk were excluded, the incidence of clinically relevant bleeding was comparable in the lower- and higher-dose osocimab and placebo groups [2]. Another phase 2 study of the FXI inhibitor fesomersen also revealed a bleeding risk similar to that of placebo [3], thus confirming the safety profile of FXI inhibition in ESKD. However, the challenge remains to demonstrate that FXI inhibition can fulfil the unmet needs in HD, including prevention of stroke in AF, reduction of adverse cardiovascular events and inhibition of clotting induced by foreign surfaces.

The conundrum of oral anticoagulation for stroke prevention in HD remains largely unresolved [4]. While non-vitamin K antagonist anticoagulants (NOACs) have a superior benefit–risk profile compared with vitamin K antagonists, their bleeding complication rate remains undeniably high and superior efficacy versus no anticoagulation has not been unequivocally demonstrated in ESKD. The CONVERT trial included patients with AF who were not considered candidates for therapeutic anticoagulation by their treating physician but was not designed to test the ability of osocimab to reduce the risk of thromboembolic events in AF.

An increasing body of evidence supports a role of NOACs in the prevention of cardiovascular disease beyond their anticoagulant properties [5]. The ongoing TRACK trial (NCT03969953) is investigating whether low-dose rivaroxaban may prevent cardiovascular events in patients with advanced CKD. Whether FXI inhibitors attenuate the inflammatory processes involved in atherosclerosis progression is unclear. In the CONVERT trial, the incidence of major adverse vascular events, a prespecified exploratory outcome, was too low to draw meaningful conclusions. Likewise, the rate of arteriovenous fistula or graft thrombosis was low and not significantly different between the groups. However, osocimab significantly reduced dialysis circuit clotting compared with placebo [2]. Similarly, another FXI inhibitor, AB023, reduced dialyzer clotting during heparin-free HD [6].

Taken together, the CONVERT trial demonstrates that FXI inhibition is a promising strategy to provide safe anticoagulation in ESKD. Other phase 2 trials are currently ongoing (Table 1). However, before we call out the discovery of the philosopher's stone with the power to cure all ailments in ESKD, large efficacy trials need to be conducted in this notoriously difficult population.
